# *Pseudomonas aeruginosa* differentially influences antibiotic-resistant *Staphylococcus aureus* emergence and expansion in hyperglycemic environments

**DOI:** 10.1128/jb.00333-25

**Published:** 2025-10-16

**Authors:** Benjamin P. Darwitz, Zachary J. Lifschin, Claire M. Miller, Christopher J. Genito, Casei A. Gossett, Kyla E. Augustine, Lance R. Thurlow

**Affiliations:** 1 Department of Microbiology and Immunology, School of Medicine, University of North Carolina at Chapel Hill 2331, , Chapel Hill, North Carolina, USA; 2 Department of Biomedical Sciences, Adams School of Dentistry, University of North Carolina at Chapel Hill 2331, Chapel Hill, North Carolina, USA; 3 Department of Nutrition, Gillings School of Global Public Health, University of North Carolina at Chapel Hill 2331, Chapel Hill, North Carolina, USA; University of Illinois Chicago, Chicago, Illinois, USA

**Keywords:** antimicrobial resistance, MRSA, infectious disease

## Abstract

Individuals with poorly controlled diabetes mellitus often develop multispecies skin and soft tissue infections, with *Staphylococcus aureus* and *Pseudomonas aeruginosa* among the most prevalent bacteria isolated from infection sites worldwide. Diabetic infections are recalcitrant to conventional antibiotic regimens and may be a reservoir for emergent antibiotic-resistant bacterial strains. Supporting this, we have previously shown that rifampicin treatment elicits the emergence and expansion of rifampicin-resistant (Rif-r) *S. aureus* only in diabetic mice, potentially due to greater bacterial outgrowth increasing the frequency of resistance-conferring mutations. However, whether *S. aureus* exhibits altered resistance outcomes during multispecies diabetic infections is unclear. During co-infection with *P. aeruginosa* under normoglycemic conditions, *S. aureus* exhibits reduced growth and altered susceptibility to several antibiotics. In contrast, we previously observed that glucose availability allows *S. aureus* to largely overcome *P. aeruginosa*-mediated growth inhibition. Here, we explored *S. aureus* resistance outcomes under hyperglycemic conditions in the context of co-infection with *P. aeruginosa* during antibiotic challenge. We found that *P. aeruginosa* exoproducts regulated by the *Pseudomonas* quinolone signal quorum sensing system inhibit the emergence but not the expansion of Rif-r *S. aureus in vitro* under glucose-replete conditions. In contrast, we recovered equivalent Rif-r *S. aureus* burdens from diabetic mice during mono- and co-infection with *P. aeruginosa*. These results demonstrate that the diabetic infection microenvironment is conducive to emergent Rif-r *S. aureus* despite external pressures elicited by *P. aeruginosa*.

**IMPORTANCE:**

Poorly controlled diabetes mellitus confers an increased susceptibility to bacterial infections, with *Staphylococcus aureus* and *Pseudomonas aeruginosa* frequently isolated from diabetic skin wounds. *S. aureus* readily develops antibiotic resistance during diabetic mono-infection under antibiotic pressure, but whether this occurs during diabetic co-infection is unclear. Under normoglycemic conditions, secreted *P. aeruginosa* factors alter *S. aureus* tolerance to several antibiotics. Here, we show that these *P. aeruginosa* exoproducts further inhibit the emergence of antibiotic-resistant *S. aureus* regardless of glucose availability *in vitro*, but this does not occur during subcutaneous co-infection in diabetic mice. These results provide initial insights regarding conditions that may inhibit *S. aureus* resistance development in hyperglycemic environments but underscore the influence of the host infection microenvironment in shaping resistance outcomes.

## INTRODUCTION

The prevalence of diabetes mellitus (DM) continues to rise in the United States, with over 11% of adults diagnosed in 2021 and an additional 38% considered to be pre-diabetic (https://www.cdc.gov/diabetes/php/data-research/index.html). Poorly controlled DM results in numerous systemic health complications, including hyperglycemia, immune suppression, and impaired wound healing, that ultimately confer a greater susceptibility to skin and soft tissue infections (SSTIs), including diabetic foot infections (DFIs) (1–5). Pathogens established in diabetic skin wounds often adopt biofilm-associated lifestyles that further impair wound healing and reduce the efficacy of conventional antibiotic therapeutics (4–6). Consequently, chronic diabetic SSTIs frequently necessitate amputation of the affected limb to prevent systemic infection (7, 8). DFIs are now the leading cause of non-traumatic amputation in the United States, with 5-year post-amputation mortality rates approaching 60% (9, 10).

Concomitant with the rising global incidence of DM is the expanding population of antimicrobial-resistant and multidrug-resistant (MDR) bacterial pathogens (11). Individuals with DM are disproportionately affected, exhibiting a higher incidence of infections with MDR pathogens than non-diabetic individuals, which is further compounded by higher antibiotic treatment rates that additionally increase the risk of selecting for resistant strains (12, 13). *Staphylococcus aureus* is the most frequently isolated bacterial pathogen from diabetic SSTIs worldwide (14–16) and is among the most prominent MDR pathogens, owing to its high virulence potential and the dwindling pool of effective treatment options (17). The diabetic wound environment is highly conducive to *S. aureus* colonization and proliferation, as hyperglycemia and immune dysfunction enhance its growth and virulence potential (18, 19). Concerningly, the combined effects of immune dysfunction, hyperglycemia, and increased rates of antibiotic treatment potentially cause the diabetic infection microenvironment to become a significant reservoir for emerging antibiotic-resistant bacterial strains (20–23). Supporting this, we have previously shown that *S. aureus* readily acquires *de novo* antibiotic resistance during subcutaneous infection in diabetic mice, which does not occur in non-diabetic controls (22). However, whether *S. aureus* differentially acquires antibiotic resistance in polymicrobial diabetic infections, which account for a significant portion of DFIs (4, 24, 25), is sorely understudied.

Alongside *S. aureus*, *Pseudomonas aeruginosa* is one of the most prevalent bacteria isolated from DFIs (14–16). The interspecies dynamics between *S. aureus* and *P. aeruginosa* have been extensively studied outside the context of diabetic infections, as both pathogens frequently cause pulmonary infection in individuals with muco-obstructive airway diseases (26). Under normoglycemic conditions, *S. aureus* growth is strongly inhibited by numerous toxins secreted by *P. aeruginosa*, encompassing LasA protease, biosurfactants, siderophores, and numerous aerobic respiration inhibitors (27–30). Notably, we have demonstrated that *S. aureus* growth inhibition caused by *P. aeruginosa* toxins is mitigated in the presence of glucose, including during co-infection of diabetic mice (31). Several of the antistaphylococcal factors produced by *P. aeruginosa* have also been shown to modulate the sensitivity of *S. aureus* to distinct antibiotics both *in vitro* and *in vivo*, such as 2-heptyl-4-hydroxyquinoline *N*-oxide (HQNO), pyocyanin, and hydrogen cyanide (HCN) (32–34). However, whether *P. aeruginosa* differentially influences the ability of *S. aureus* to acquire antibiotic resistance within the diabetic infection microenvironment remains unknown. Understanding how interspecies interactions between bacterial pathogens influence antibiotic treatment outcomes within hyperglycemic environments may reveal novel mechanisms regarding the evolution of antibiotic resistance in diabetic infections.

In this study, we found that secreted *P. aeruginosa* antistaphylococcal compounds inhibited the emergence of a resistant *S. aureus* subpopulation under rifampicin (Rif) pressure *in vitro*, including within hyperglycemic conditions. In contrast, we show that *S. aureus* growth is attenuated during co-infection with *P. aeruginosa* in diabetic mice; however, this attenuation does not prevent the acquisition of Rif resistance during treatment. Altogether, our findings demonstrate that co-infection conditions can modulate *S. aureus* antibiotic resistance acquisition within hyperglycemic environments *in vitro* but underscore that the host infection microenvironment likewise influences the dynamics of antibiotic resistance emergence in *S. aureus* during co-infection with *P. aeruginosa*.

## RESULTS

### *P. aeruginosa* exoproducts inhibit the emergence and expansion of rifampicin-resistant *S. aureus* under hyperglycemic conditions

We previously demonstrated that *S. aureus* readily acquires resistance to the antibiotic Rif during subcutaneous infection in diabetic mice under antibiotic pressure, whereas resistance was never observed in non-diabetic controls (22). Although Rif is not routinely used to treat *S. aureus* SSTIs in clinical practice, we selected it for our studies due to the high propensity of *S. aureus* to develop resistance in permissive environments and its widespread use as a tool for quantifying bacterial mutation frequencies (26, 35, 36). The differential emergence and expansion of rifampicin-resistant (Rif-r) *S. aureus* populations in diabetic mice may reflect the enhanced proliferative capacity of *S. aureus* within the diabetic infection microenvironment, which increases the likelihood of acquiring *de novo* point mutations conferring high-level Rif resistance (3, 18, 22). Separately, we have shown that *S. aureus* outgrowth is moderately attenuated during co-infection with *P. aeruginosa* relative to mono-infection in diabetic mice (31). Based on these prior observations, we hypothesized that *P. aeruginosa*-mediated growth inhibition would likewise suppress the emergence or otherwise limit the expansion of Rif-r *S. aureus* under hyperglycemic conditions. To test this *in vitro*, we grew Rif-sensitive *S. aureus* in cell-free *Staphylococcus aureus* supernatants (SA sup) or *Pseudomonas aeruginosa* supernatants (PA sup), respectively, supplemented with either 1% casamino acid (CAA) plus 25 mM dextrose (Dex) or a carbon-equivalent concentration of CAA alone (1.892%). Cultures were treated with 0.8 µg/mL Rif (100× the MIC for *S. aureus*) or left untreated, and both total and Rif-r *S. aureus* CFU were quantified at 6 and 24 h following Rif treatment (T6 and T24, respectively). Supernatants were used instead of live co-culture to isolate the effects of secreted *P. aeruginosa* exoproducts independent of direct bacterial competition for resources. We used SA sup as a control medium to account for nutritional differences between the supernatants relative to the base medium that may have resulted from bacterial growth and supernatant conditioning.

**Fig 1:**
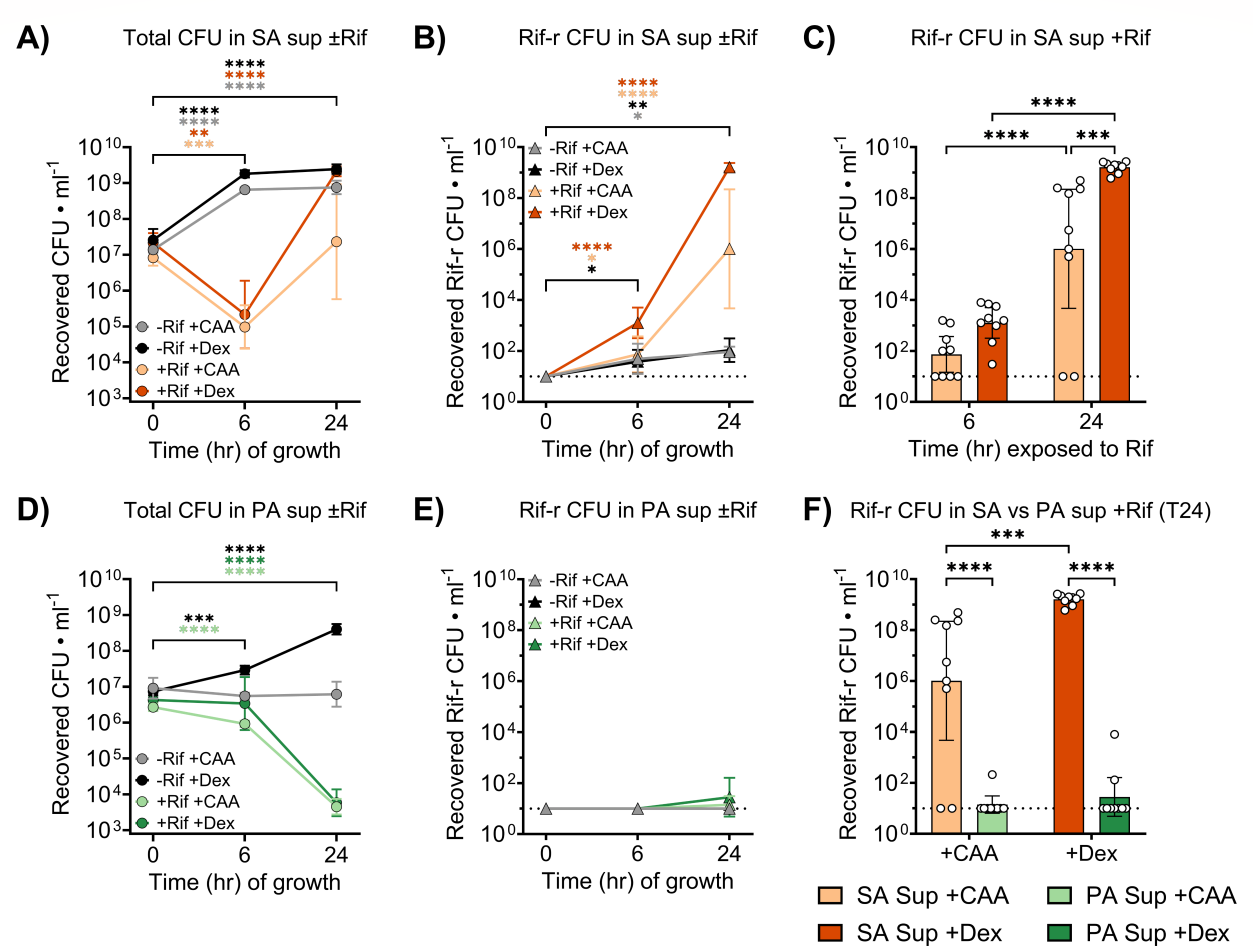
*S. aureus* readily acquires rifampicin (Rif) resistance in *Staphylococcus aureus* supernatants (SA sup) supplemented with dextrose (Dex) but not in *Pseudomonas aeruginosa* supernatants (PA sup). (**A**) Total and (**B**) rifampicin-resistant (Rif-r) *S. aureus* burdens were quantified from SA sup supplemented with casamino acid (CAA) or Dex, following 0, 6, and 24 h of incubation with or without Rif. (**C**) Comparison of Rif-r *S. aureus* burdens in Rif-treated SA sup supplemented with CAA or Dex at T6 and T24. (**D**) Total and (**E**) Rif-r *S. aureus* burdens were quantified from PA sup supplemented with CAA or Dex, following 0, 6, and 24 h of incubation with or without Rif. (**F**) Comparison of Rif-r *S. aureus* burdens in Rif-treated SA sup versus PA sup supplemented with CAA or Dex at T24. Points and bars represent geometric means and 95% confidence interval. *P < 0.05, **P < 0.01, ***P < 0.001, ****P < 0.0001; two-way analysis of variance with Dunnett’s multiple comparisons to T0 (**A–E**) or Sidak’s multiple comparisons between groups differing by a single experimental factor (**C, F**). The dotted line represents the limit of detection.

In SA sup, Rif treatment induced a transient decline in total *S. aureus* burden at T6 compared to T0 (Fig. 1A), which was followed by substantial regrowth between T6 and T24, especially in Dex-supplemented conditions (Fig. S1A). This growth resurgence coincided with an expanding Rif-r *S. aureus* subpopulation, which was first detected at T6 and continued to grow through T24 (Fig. 1B). By T24, Rif-r burden was significantly higher in Dex-supplemented SA sup than in CAA-only media (Fig. 1C) and strongly correlated with total CFU (Fig. S1B), indicating that population recovery was being driven by the expansion of the Rif-r subpopulation under antibiotic pressure. These results align with previous findings showing that Dex supplementation supported the expansion of Rif-r *S. aureus* during Rif challenge in tryptic soy broth (TSB) (22).

In contrast, *S. aureus* cultured in Rif-treated PA sup exhibited substantial population decline by T24 in both Dex- and CAA-supplemented media (Fig. 1D), which was accompanied by an overall negligible recovery of Rif-r colonies (Fig. 1E). At T24, both total (Fig. S1C) and Rif-r (Fig. 1F) burdens were significantly lower than those in SA sup, regardless of carbon source (*P* < 0.0001 for both comparisons). Notably, 15 of the 18 PA sup samples lacked any detectable Rif-r isolates (Fig. S1D), contrasting our observations from Dex-supplemented SA sup, where Rif-r *S. aureus* always arose as the predominant (>50%) subpopulation during Rif treatment (Fig. S1E). Altogether, these preliminary data suggested that secreted *P. aeruginosa* factors were inhibiting the emergence or otherwise limiting the expansion of Rif-r *S. aureus* in hyperglycemic conditions.

### The *Pseudomonas* quinolone signal quorum sensing system of *P. aeruginosa* is integral to limiting rifampicin-resistant *S. aureus* emergence and expansion

To begin characterizing the *P. aeruginosa* factors responsible for suppressing the Rif-r subpopulation, we next tested whether *S. aureus* could acquire resistance when cultured in PA sup depleted of known antistaphylococcal factors. *P. aeruginosa* virulence is tightly regulated by its interconnected quorum sensing (QS) systems: Las, Rhl, and *Pseudomonas* quinolone signal (PQS) (37). Notably, these QS systems control the production of several respiration inhibitors that potently inhibit *S. aureus* growth, including HQNO (28), pyocyanin (29), and HCN (30). Previous studies have shown that disruption of the PQS system significantly reduces the production of these respiration inhibitors (38–41). Consistent with these findings, we have demonstrated that *S. aureus* growth is restored in PA sup derived from a PQS-deficient mutant (Δ*pqsR*) (31), which lacks the master regulator of the PQS system (42). Therefore, we repeated our Rif challenge assays using supernatants harvested from Δ*pqsR* cultures.

**Fig 2:**
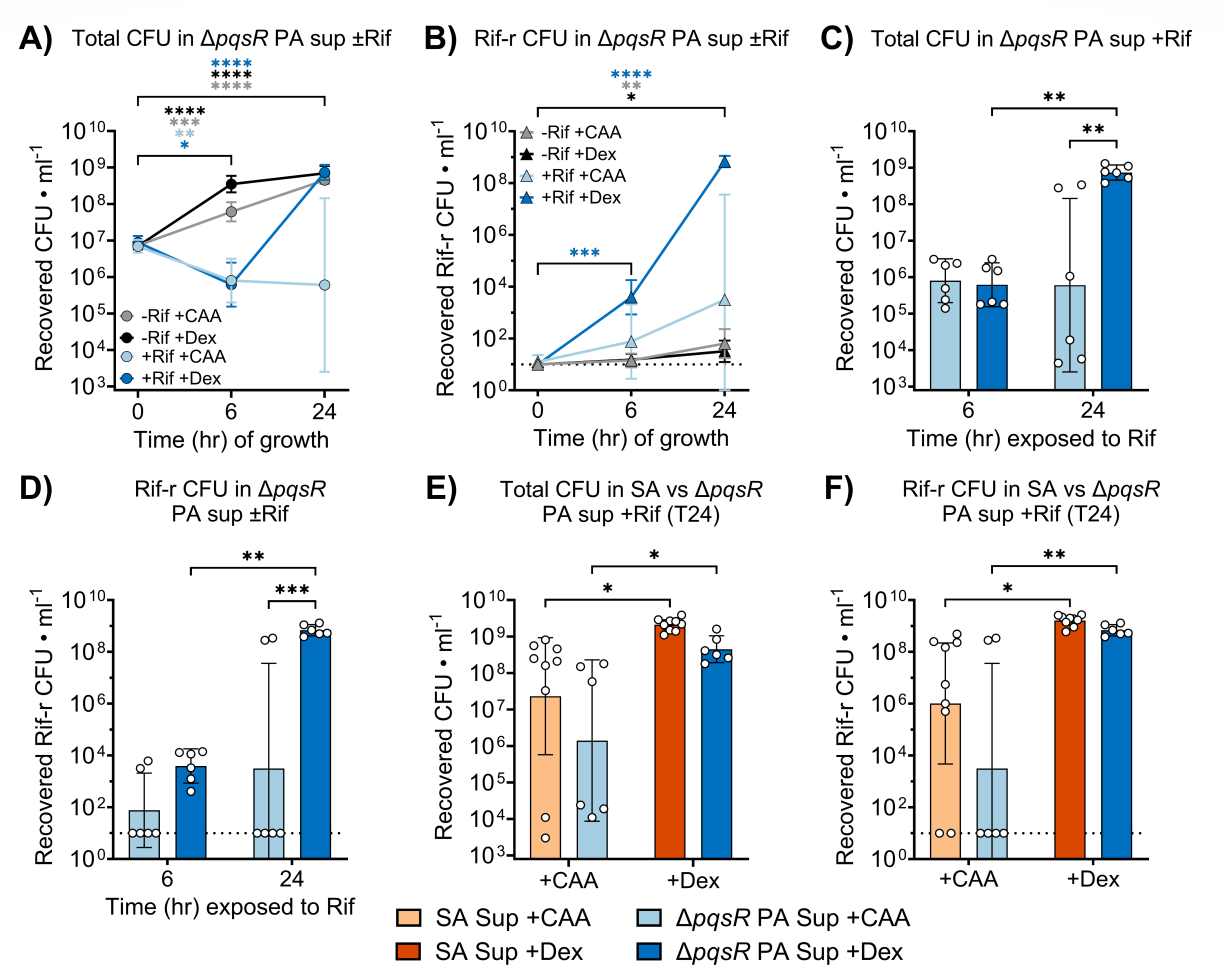
*S. aureus* retains the ability to acquire rifampicin (Rif) resistance in *P. aeruginosa* supernatants harvested from a *Pseudomonas* quinolone signal-deficient mutant. (**A**) Total and (**B**) rifampicin-resistant (Rif-r) *S. aureus* burdens were quantified from Δ*pqsR P. aeruginosa* supernatants (Δ*pqsR* PA sup) supplemented with casamino acid (CAA) or dextrose (Dex), following 0, 6, and 24 h of incubation with or without Rif. Comparison of (**C**) total and (**D**) Rif-r *S. aureus* burdens in Rif-treated Δ*pqsR* PA sup supplemented with CAA or Dex at T6 and T24. Comparison of (**E**) total and (**F**) Rif-r *S. aureus* burdens in Rif-treated SA sup versus Rif-treated Δ*pqsR* PA sup at T24 with CAA or Dex. Points and bars represent geometric means and 95% confidence intervals. *P < 0.05, **P < 0.01, ***P < 0.001, ****P < 0.0001; two-way analysis of variance with Dunnett’s multiple comparisons to T0 (**A and B**) or Sidak’s multiple comparisons between groups differing by a single experimental factor (**C–F**). The dotted line represents the limit of detection.

Similar to what was observed in SA sup, total *S. aureus* burden in Rif-treated Δ*pqsR* PA sup initially declined from T0 to T6 (Fig. 2A) but rebounded from T6 to T24 in Dex-supplemented media (Fig. 2C). This recovery coincided with the emergence and expansion of a Rif-r subpopulation, first detected at T6 (Fig. 2B) and increasing by T24 (Fig. 2D). *S. aureus* exhibited minor growth inhibition in untreated Δ*pqsR* PA sup at both T6 (Fig. S2A) and T24 (Fig. S2B) compared to SA sup, in contrast to the substantial growth suppression observed in untreated wild-type (WT) PA sup relative to Δ*pqsR* PA sup (Fig. S2C through E). During Rif treatment, total *S. aureus* burden at T24 did not significantly differ between SA sup and Δ*pqsR* PA sup (Fig. 2E), and comparable numbers of Rif-r colonies were recovered from both conditions, regardless of carbon source (Fig. 2F). Together, these findings provide compelling evidence that PQS-regulated factors produced by *P. aeruginosa* inhibit the emergence of Rif-resistant *S. aureus* by limiting overall population expansion.

### Glucose availability promotes the expansion of rifampicin-resistant *S. aureus* isolates despite increased susceptibility to *P. aeruginosa* toxins

We next investigated whether secreted *P. aeruginosa* factors exhibited differential toxicity to select Rif-r isolates, thus preventing their expansion. Specific point mutations in *rpoB*, which encodes the β-subunit of RNA polymerase, confer Rif resistance in *S. aureus* but can carry various fitness costs (43, 44). For example, the His481Tyr substitution in RpoB confers heightened sensitivity to oxidative stress (45, 46), which may be exacerbated by *P. aeruginosa* respiration inhibitors (26, 47). Conversely, the Ser486Leu substitution is associated with a slightly lower growth rate compared to its parental WT strain but has not been linked to increased susceptibility to oxidative stress (43, 48). Based on these observations, we reasoned that measuring the growth of distinct Rif-r *S. aureus* isolates in PA sup would allow us to determine whether *P. aeruginosa* exoproducts limit Rif-r subpopulation expansion by potentiating susceptibilities to stress-related toxicity or otherwise exacerbating known fitness defects. To test this, we compared the growth of *S. aureus* RpoB(Ser486Leu) and RpoB(His481Tyr) strains (hereafter referred to as SA-S486L and SA-H481Y, respectively) in SA and PA sup relative to WT *S. aureus*. If either mutant failed to expand in PA sup, it would suggest that secreted *P. aeruginosa* toxins suppress the growth of select Rif-r isolates, irrespective of their emergence.

**Fig 3:**
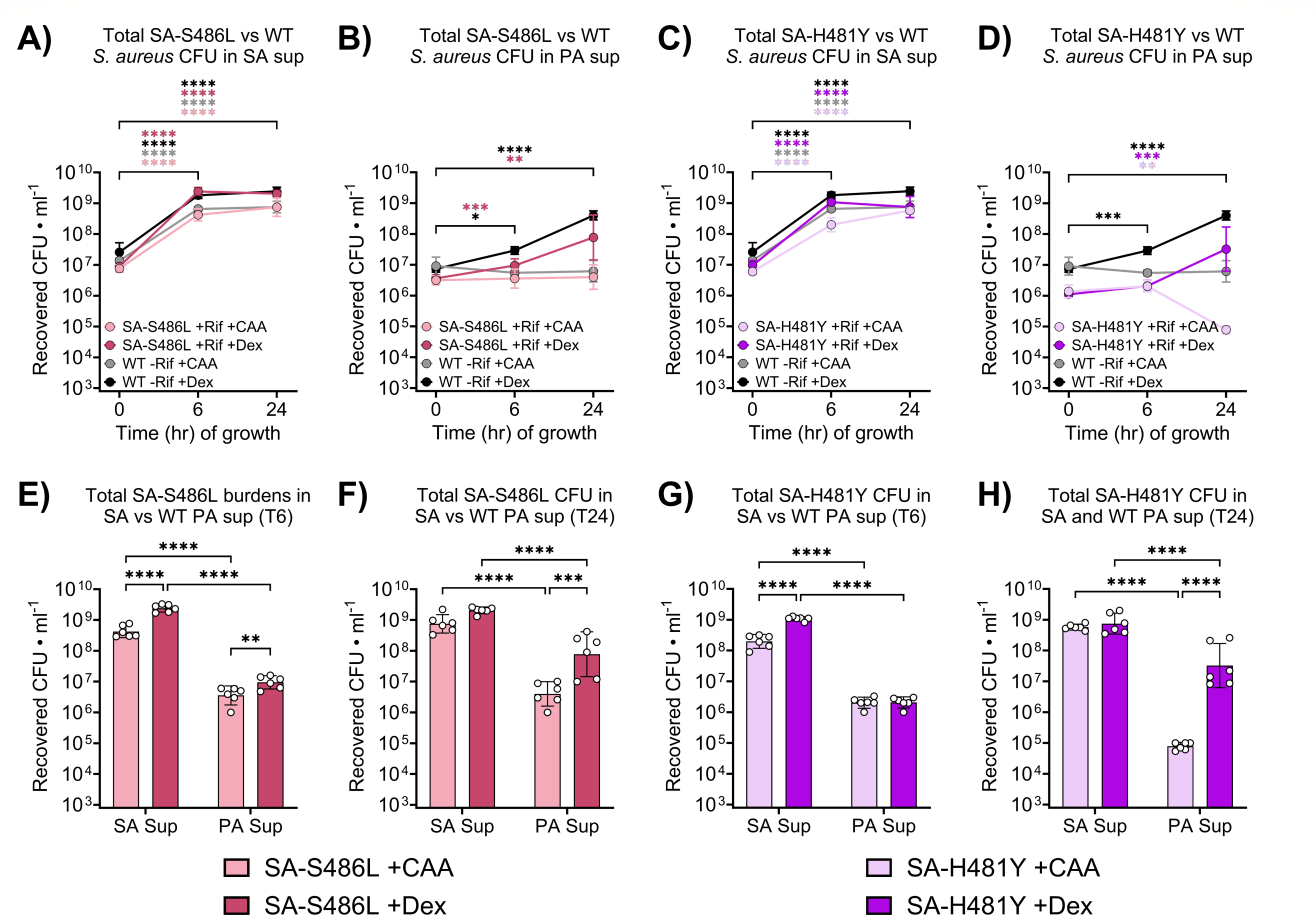
*P. aeruginosa* supernatants (PA sup) selectively inhibit the expansion of rifampicin-resistant (Rif-r) *S. aureus* isolates but not in hyperglycemic conditions. Growth of WT and SA-S486L *S. aureus* in (**A**) *S. aureus* supernatants (SA sup) and (**B**) PA sup supplemented with casamino acid (CAA) or dextrose (Dex), following 0, 6, and 24 h of incubation. Growth of WT and SA-H481Y *S. aureus* in (**C**) SA sup and (**D**) PA sup supplemented with CAA or Dex, following 0, 6, and 24 h of incubation. Comparison of SA-S486L burdens recovered from SA sup versus PA sup at (**E**) T6 and (**F**) T24 with CAA or Dex. Comparison of SA-H481Y burdens recovered from SA sup versus PA sup at (**G**) T6 and (**H**) T24 with CAA or Dex. Points and bars represent geometric means and 95% confidence intervals. *P < 0.05, **P < 0.01, ***P < 0.001, ****P < 0.0001; two-way analysis of variance with Dunnett’s multiple comparisons to T0 (**A–D**) or Sidak’s multiple comparisons between groups differing by a single experimental factor (**E–H**).

Aligning with our results using WT *S. aureus*, SA-S486L exhibited robust growth in SA sup under both carbon conditions (Fig. 3A) and only expanded in PA sup supplemented with Dex (Fig. 3B), with recovered burdens consistently higher in SA sup compared to PA sup at both T6 (Fig. 3E) and T24 (Fig. 3F). Additionally, we did not observe a substantial growth defect in SA-S486L relative to WT *S. aureus* under most tested conditions (Fig. S3). Given that SA-S486L was able to expand in Dex-supplemented PA sup, we concluded that secreted *P. aeruginosa* factors do not universally prevent the expansion of Rif-r isolates, supporting the notion that these factors are inhibiting initial resistance emergence.

Unlike SA-S486L, SA-H481Y exhibited slight growth defects in SA sup relative to WT *S. aureus* (Fig. S3A and B). Although SA-H481Y expanded in both CAA- and Dex-supplemented SA sup (Fig. 3C), recovered burdens from PA sup were consistently lower than in SA sup across all experimental timepoints and conditions (Fig. 3G and H). By T24, SA-H481Y grew in Dex-supplemented PA sup but exhibited population decline in PA sup with CAA alone over the same time frame (Fig. 3D), potentially due to the increased sensitivity to oxidative stress previously described in this mutant (46). These findings suggest that *P. aeruginosa* exoproducts not only inhibit the emergence of Rif-r isolates but may also selectively restrict the expansion of stress-sensitive strains. However, the ability of SA-H481Y to expand in Dex-supplemented PA sup suggests that hyperglycemic conditions permit the outgrowth of resistant *S. aureus* strains despite stress-related vulnerabilities.

### *P. aeruginosa* does not inhibit the emergence or expansion of rifampicin-resistant *S. aureus* in murine subcutaneous co-infections

**Fig 4:**
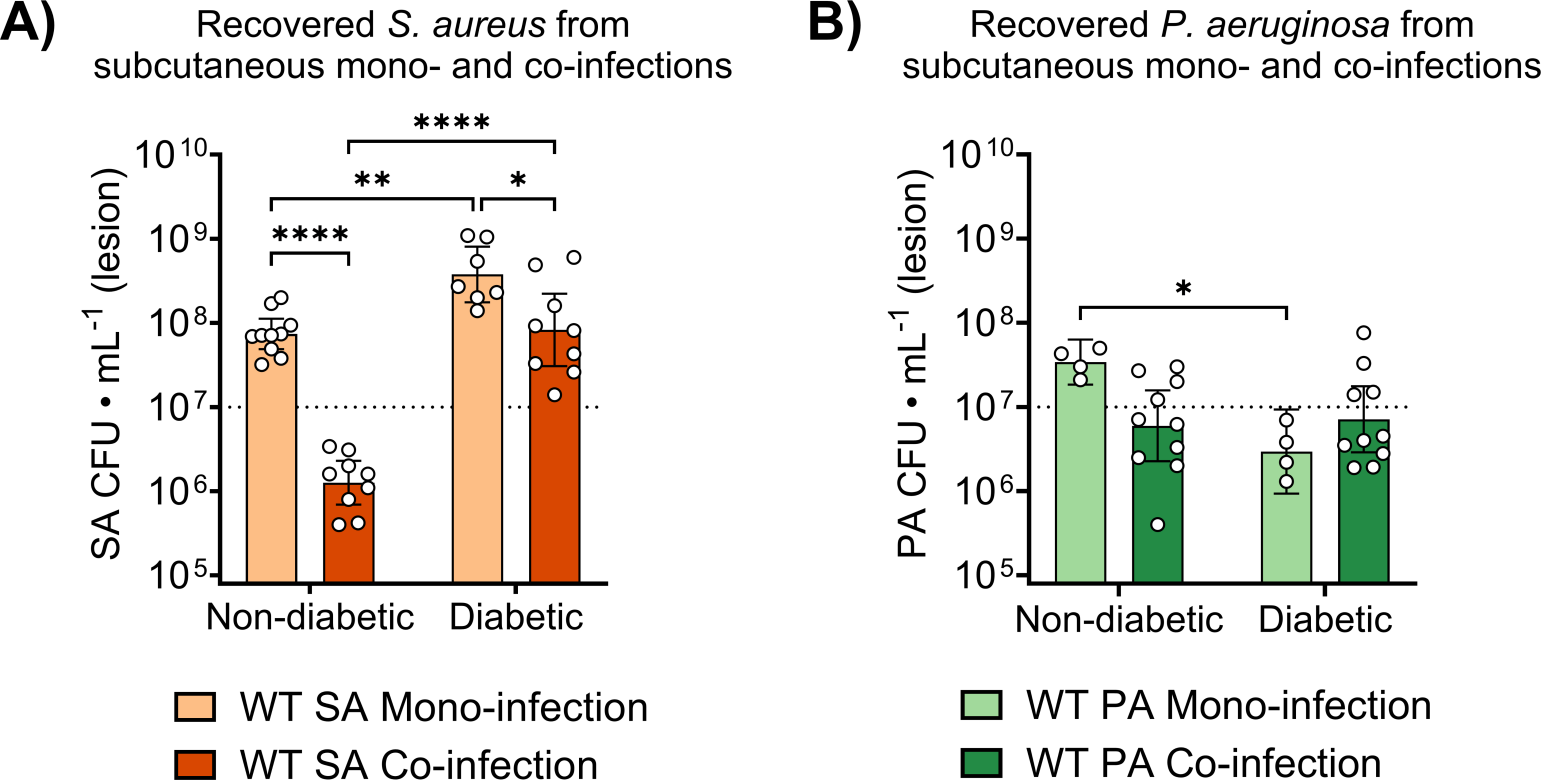
*S. aureus* growth is attenuated during subcutaneous co-infection with *P. aeruginosa* in non-diabetic and diabetic mice, with comparable growth observed during non-diabetic mono-infection and diabetic co-infection. Total (**A**) *S. aureus* and (**B**) *P. aeruginosa* burdens recovered from the lesions of non-diabetic and diabetic mice during subcutaneous mono- or co-infection. Bars represent geometric means and 95% confidence intervals. *P < 0.05, **P < 0.01, ****P < 0.0001; two-way analysis of variance with Sidak’s multiple comparisons between groups differing by a single experimental factor. Dotted lines represent infection inoculum.

Although *P. aeruginosa* exoproducts appeared to suppress Rif-r *S. aureus* emergence and expansion *in vitro*, it remained unclear whether this inhibitory effect would persist *in vivo*, where host-derived factors contribute to bacterial heterogeneity and often drive behaviors distinct from those seen under *in vitro* conditions (47). We have previously shown that *S. aureus* growth is attenuated during co-infection with *P. aeruginosa* in a subcutaneous catheter co-infection model, even in diabetic mice (31). These previous results agree with data derived from the subcutaneous co-infection model implemented in this study, wherein we recovered ~1 log lower *S. aureus* burden from untreated diabetic mice co-infected with *P. aeruginosa* compared to mono-infection (Fig. 4A). Furthermore, there was no significant difference in *S. aureus* burdens from co-infected diabetic mice compared to mono-infected non-diabetic mice (Fig. 4A). Because we previously did not observe an expanding Rif-r population in Rif-treated non-diabetic mice (22), we reasoned that the growth inhibition observed in diabetic mice during co-infection may result in limited emergence of Rif-r isolates by constraining *S. aureus* population size. We therefore sought to interrogate whether *P. aeruginosa* restricts the emergence and expansion of Rif-r *S. aureus* in our murine subcutaneous co-infection model. To perform our investigation, diabetic mice and non-diabetic controls were infected with Rif-sensitive *S. aureus* alone or in combination with *P. aeruginosa* and treated with Rif for four consecutive days. Total and Rif-r *S. aureus* were then quantified from lesion homogenates. Additionally, *P. aeruginosa* exhibits a high degree of intrinsic resistance to Rif (49), which allowed us to directly assess the combined effects of Rif treatment and *P. aeruginosa* co-infection on *S. aureus* populations.

**Fig 5:**
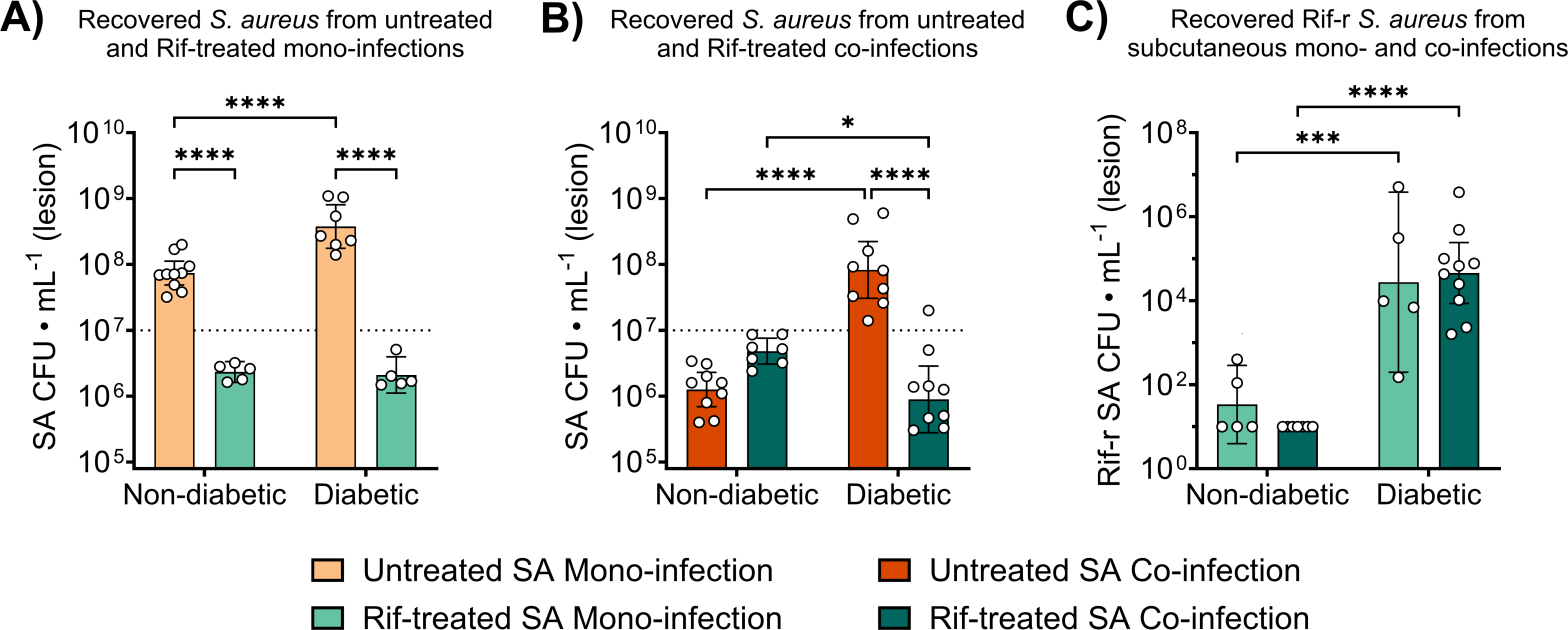
*P. aeruginosa* does not inhibit the development of rifampicin (Rif) resistance in *S. aureus* during non-diabetic and diabetic co-infections. Total *S. aureus* burdens from untreated and Rif-treated non-diabetic and diabetic mice were quantified during (**A**) mono-infection or (**B**) co-infection with *P. aeruginosa*. (**C**) Rifampicin-resistant (Rif-r) *S. aureus* was enumerated from Rif-treated non-diabetic and diabetic mice during mono-infection or co-infection with *P. aeruginosa*. Bars represent geometric means and 95% confidence intervals. *P < 0.05, ***P < 0.001, ****P < 0.0001; two-way analysis of variance with Sidak’s multiple comparisons between groups differing by a single experimental factor. Dotted lines represent infection inoculum.

Consistent with previous results (22), total *S. aureus* burdens were lower in Rif-treated animals during mono-infection regardless of diabetic status (Fig. 5A), demonstrating that rifampicin was effective for reducing overall *S. aureus* burdens when it was the sole infecting organism. During co-infection, we recovered slightly lower total *S. aureus* burdens from Rif-treated diabetic mice compared to their non-diabetic counterparts (Fig. 5B). Contrary to our hypothesis that *P. aeruginosa* would suppress the expansion of Rif-r *S. aureus* in diabetic co-infections, we recovered equivalent Rif-r burdens from mono- and co-infected mice following antibiotic treatment, despite the overall reduction in *S. aureus* burden observed during untreated co-infection (Fig. 5C). *P. aeruginosa* burdens remained stable across co-infection groups (Fig. S4), ensuring that the observed differences in *S. aureus* populations were not due to variances in *P. aeruginosa* burden. Altogether, these data highlight how the host environment can shape the interspecies dynamics between co-infecting bacteria and may even override strong effects observed under *in vitro* models.

## DISCUSSION

We have previously demonstrated that *S. aureus* readily acquires Rif resistance under antibiotic pressure in diabetic mice, likely due to its expanded proliferation increasing the frequency of Rif-r emergence (22). Additionally, we showed that the bacterium resists *P. aeruginosa*-mediated growth inhibition under hyperglycemic conditions (31). However, it remains unclear whether *P. aeruginosa* modulates the ability of *S. aureus* to acquire antibiotic resistance within this permissive environment. Here, we show that *P. aeruginosa* exoproducts inhibit both the emergence and expansion of Rif-r *S. aureus in vitro* under hyperglycemic conditions (Fig. 1F) and that the PQS quorum sensing system of *P. aeruginosa* is integral for maintaining this phenomenon (Fig. 2D). The PQS system controls the production of several aerobic respiration inhibitors, including HQNO, pyocyanin, and HCN, that both inhibit components of the *S. aureus* electron transport chain (ETC) and generate reactive oxygen species (26, 42, 50–52). Under glucose-replete conditions, *S. aureus* may mitigate these effects through overflow metabolism, which involves redirecting pyruvate concentrations exceeding TCA cycle capacity through fermentative pathways to generate ATP in a redox-balanced manner under aerobic conditions (53). This process of metabolic reprogramming reduces *S. aureus* dependency on aerobic respiration in hyperglycemic environments (54) and may explain both the modest recovery of *S. aureus* growth observed in Dex-supplemented PA sup and the complete growth arrest that occurred in untreated CAA-only PA sup (Fig. 1D; Fig. S1F). These findings are consistent with previous work showing that *S. aureus* mutants without functional ETC fail to grow in CAA-only media and display reduced growth when glucose is available (55).

Because mutations conferring Rif-resistance arise during DNA replication (56), we hypothesized that it was unlikely *S. aureus* would acquire *de novo* Rif resistance in environments that inhibited population expansion. Indeed, *S. aureus* failed to expand in untreated CAA-supplemented PA sup over 24 h, which coincided with the recovery of Rif-r *S. aureus* from only a single PA sup + CAA sample during Rif treatment (Fig. 1F; Fig. S1D). However, this does not explain the lack of Rif-r *S. aureus* recovered from Rif-treated PA sup + Dex. Although *S. aureus* grew in Dex-supplemented PA sup in the absence of Rif (Fig. 1D; Fig. S1F), Rif-r colonies were detected in only two samples across treatment conditions (Fig. 1F; Fig. S1D). This was unexpected, given that *S. aureus* populations in Dex-supplemented PA sup reached levels that should have supported a detectable population of emergent Rif-r mutants, even without clonal expansion (22). Because the Rif-r isolates SA-S486L and SA-H481Y both grew in Dex-supplemented PA sup (Fig. 3B and D), we concluded that it was unlikely that Rif-r isolates were emerging but not expanding to detectable levels in PA sup. Interestingly, previous work shows that *S. aureus* exhibits increased mutation frequency when exposed to PQS-regulated compounds (57), which contrasts the absence of Rif-r isolates from Dex-supplemented PA sup in our experiments. Furthermore, *P. aeruginosa* exoproducts readily induce conditions that can activate the distress signal SOS response in *S. aureus* (58), which has been directly linked to increased mutation rates (59), including those conferring Rif resistance (36). Our results contradicting these findings suggest that PQS-regulated exoproducts may interfere with resistance emergence through mechanisms beyond alterations in mutation frequency. Indeed, even the spontaneous emergence of Rif-r mutants in the absence of Rif pressure was abrogated by PA sup (Fig. 1E).

Despite the potent inhibitory effects of PQS-regulated *P. aeruginosa* exoproducts on the emergence of resistant *S. aureus in vitro*, comparable levels of Rif-r isolates were recovered from both mono- and co-infected diabetic mice under antibiotic pressure (Fig. 5C). This discrepancy may result from host-associated factors that limit either the production, localized distribution, or antistaphylococcal activity of *P. aeruginosa* exoproducts during infection. Notably, *P. aeruginosa* exhibits increased biofilm formation in hyperglycemic environments (60), including within diabetic SSTIs (61). Biofilm-associated *P. aeruginosa* displays altered QS dynamics that are further influenced by environmental factors, including cell density, as well as oxygen and iron availability (62). In line with these observations, *P. aeruginosa* burdens remained within 1 log of the initial inoculum in our studies (Fig. 4B; Fig. S4), suggesting that the bacterial density required for robust QS activation and toxin production may not have been achieved in our model.

One additional consideration is that *P. aeruginosa* and *S. aureus* are known to colonize distinct niches within the wound bed during mono-infection, with *S. aureus* localizing near the surface and *P. aeruginosa* residing deeper in dermis (63). This spatial segregation may be reinforced by HQNO, which has been shown to promote the separation of *P. aeruginosa* and *S. aureus* during biofilm-associated co-culture (64). As a result, the diffusion of PQS toxins within the lesion may be insufficient to exert inhibitory effects on *S. aureus*. Nevertheless, *P. aeruginosa* still appears to constrain *S. aureus* growth in diabetic co-infections (Fig. 4A), potentially due to competition for resources. This inhibitory effect was more pronounced in non-diabetic mice, where lower glucose availability may be exacerbating interspecies competition for carbon sources. Collectively, these findings suggest that the degree of *S. aureus* suppression elicited by PQS-regulated factors *in vitro* may not be fully recapitulated *in vivo*.

Given that *S. aureus* loses the enhanced growth observed in diabetic mono-infection during co-infection with *P. aeruginosa*, we were surprised to recover Rif-r isolates from diabetic co-infections but rarely from non-diabetic mono-infections (Fig. 5C). This discrepancy may reflect the 1-day delay between initial infection and antibiotic treatment, which could provide a limited window for the emergence of resistant mutants in diabetic mice, where hyperglycemia may enhance *S. aureus* replication despite inhibitory pressures from *P. aeruginosa*. Upon starting Rif treatment, susceptible *S. aureus* potentially succumbs to antibiotic pressure while pre-existing Rif-r isolates expand, albeit at a reduced rate, given the apparent constraints imposed by *P. aeruginosa* co-infection. This finding suggests that perhaps even transient opportunities for replication within the diabetic infection microenvironment can enable the emergence of antibiotic-resistant *S. aureus*, irrespective of interspecies competition.

In summary, our findings demonstrate that *P. aeruginosa* exoproducts regulated by the PQS QS system inhibit the emergence and expansion of Rif-r *S. aureus in vitro*, including under hyperglycemic conditions. However, these inhibitory effects are not fully recapitulated during diabetic co-infection, potentially due to host-specific factors that may limit both *P. aeruginosa* QS activity and interspecies interactions. These observations underscore the permissive nature of the diabetic infection microenvironment in promoting the emergence of antibiotic-resistant bacterial strains, even in the context of polymicrobial competition.

## MATERIALS AND METHODS

### Bacterial strains and growth conditions

All *S. aureus* strains used in this study were from the JE2 background, a derivative of the USA300 lineage. All *P. aeruginosa* strains originated from MPAO1, a widely used laboratory reference strain. Overnight cultures of *S. aureus* and *P. aeruginosa* were prepared using TSB without dextrose and Luria-Bertani broth, respectively, and incubated at 37°C with shaking at 250 revolutions per minute (rpm) for 18–24 h. Rif-r *S. aureus* strains SA-S486L and SA-H481Y were obtained from individual colonies isolated from *in vitro* SA sup experiments described in this study. Genomic DNA was extracted from each isolate using the Wizard Genomic DNA Purification Kit (Promega) according to the manufacturer’s protocol. The *rpoB* locus was PCR-amplified using primers rpoB-F (5′-GGTAATGCTTTCCCTGACTC-3′) and rpoB-R (5′-GCTTCGGCGATACATCC-3′) with NEBNext High-Fidelity PCR Master Mix (New England Biolabs). Point mutations in *rpoB* were identified by Sanger sequencing (Azenta Life Sciences).

### Generation of cell-free supernatants

Cell-free supernatants were prepared as described previously (31). Briefly, 500 mL Erlenmeyer flasks containing 100 mL of TSB without dextrose were inoculated with 0.5 mL of bacterial culture. Flasks were sealed with Breathe-EASIER membranes (Diversified Biotech) and incubated for 24 h at 37°C with shaking at 250 rpm. Cultures were decanted into 50 mL conical tubes and centrifuged at 3,200 × *g* for 30 min, and the resulting supernatants were decanted into fresh 50 mL conical tubes, filter sterilized through a 0.45 µm filter, and titrated to pH 7.0 with 10 N hydrogen chloride. Supernatants were supplemented with either 25 mM glucose and 1% wt/vol Bact CAA (Thermo Fisher Scientific) or with 1.892% wt/vol CAA alone, which matches the carbon content of glucose-supplemented media. The adjusted CAA concentration in CAA-only supernatants was determined based on the published amino acid profile of casamino acids (US Biological Life Sciences), further excluding amino acids not utilized as carbon sources by *S. aureus* (65). All supernatants were filtered through a 0.45 µm filter again, aliquoted, and stored at −80°C until use.

### Rifampicin kill curve studies

For each condition, 10 µL of overnight *S. aureus* culture was used to inoculate 3 mL of cell-free supernatants in triplicate, then incubated for 2 h at 37°C with shaking at 250 rpm. Following this initial incubation, samples were plated using drip and spread plate methods on tryptic soy agar (TSA) and TSA containing 3 µg/mL Rif to quantify total and Rif-r *S. aureus* CFU/mL, respectively; these counts were designated as T0. Samples were treated with either 0.8 µg/mL Rif (100× the MIC) or vehicle control (dimethyl sulfoxide) and returned to the incubator, and total and Rif-r *S. aureus* were enumerated at 6 and 24 h post-treatment. Before enumeration, Rif was removed from samples by centrifuging 200 µL aliquots at 16,000 × *g* for 1 min, decanting the supernatants, and washing the bacterial pellets twice in 200 µL sterile phosphate-buffered saline (PBS). We determined the limit of detection (LOD) for CFU enumeration to be 10 CFU/mL based on the lowest dilution series being derived from plating 100 µL of undiluted samples.

### Animal studies

All animal studies were conducted at the University of North Carolina at Chapel Hill in an accredited Association for Assessment and Accreditation of Laboratory Animal Care facility, in accordance with an Institutional Animal Care and Use Committee-approved protocol. Male C57BL/6J mice obtained from the Jackson Laboratory were used in this study. Diabetes was induced by five consecutive daily intraperitoneal (ip) injections of streptozotocin (65 mg/kg) followed by a 5-day rest period. Blood glucose levels were measured via tail snip with a glucometer, and mice with blood glucose levels ≥300 mg/dL were considered diabetic.

For infections, 20 µL of a PBS suspension containing 1 × 10^7^ CFU *S*. *aureus* and/or *P. aeruginosa* (in mono- or co-culture) was subcutaneously injected into mouse flanks. After 24 h, a subset of mice was treated with Rif (25 mg/kg, ip) once daily for four consecutive days. On day 5, resulting lesions were harvested, homogenized in 500 µL PBS, and plated via spread and drip plate methods on *Pseudomonas* Isolation Agar (Thermo Fisher Scientific), mannitol salt agar (MSA; Sigma-Aldrich), and MSA containing 3 µg/mL Rif to enumerate *P. aeruginosa*, *S. aureus*, and Rif-r *S. aureus*, respectively.

### Statistical analysis

CFU data were log_10_-transformed before statistical analysis. Samples with no detectable colonies were assigned counts corresponding to the experimental LOD. *In vitro* CFU counts were assessed using two-way analysis of variance (ANOVA) with the appropriate post-test for multiple comparisons, as denoted in the figure legends. *In vivo* data were assessed using two-way ANOVA followed by Sidak’s multiple comparison test for selected pairwise comparisons between groups differing by a single experimental factor. All statistical analyses were performed using GraphPad Prism 10.

## SUPPLEMENTAL MATERIAL

Supplemental figures jb.00333-25-s0001.pdf Figures S1 to S4.

ASM does not own the copyrights to Supplemental Material that may be linked to, or accessed through, an article. The authors have granted ASM a non-exclusive, world-wide license to publish the Supplemental Material files. Please contact the corresponding author directly for reuse.
