# Supplementary material for: Pseudomonas aeruginosa differentially influences antibiotic-resistant Staphylococcus aureus emergence and expansion in hyperglycemic environments
Source: J Bacteriol. 2025 Oct 16;207(11):e00333-25. doi: 10.1128/jb.00333-25 (PMC12632273; doi:10.1128/jb.00333-25)
Supplement: Supplemental figures — Figures S1 to S4. [file jb.00333-25-s0001.pdf]

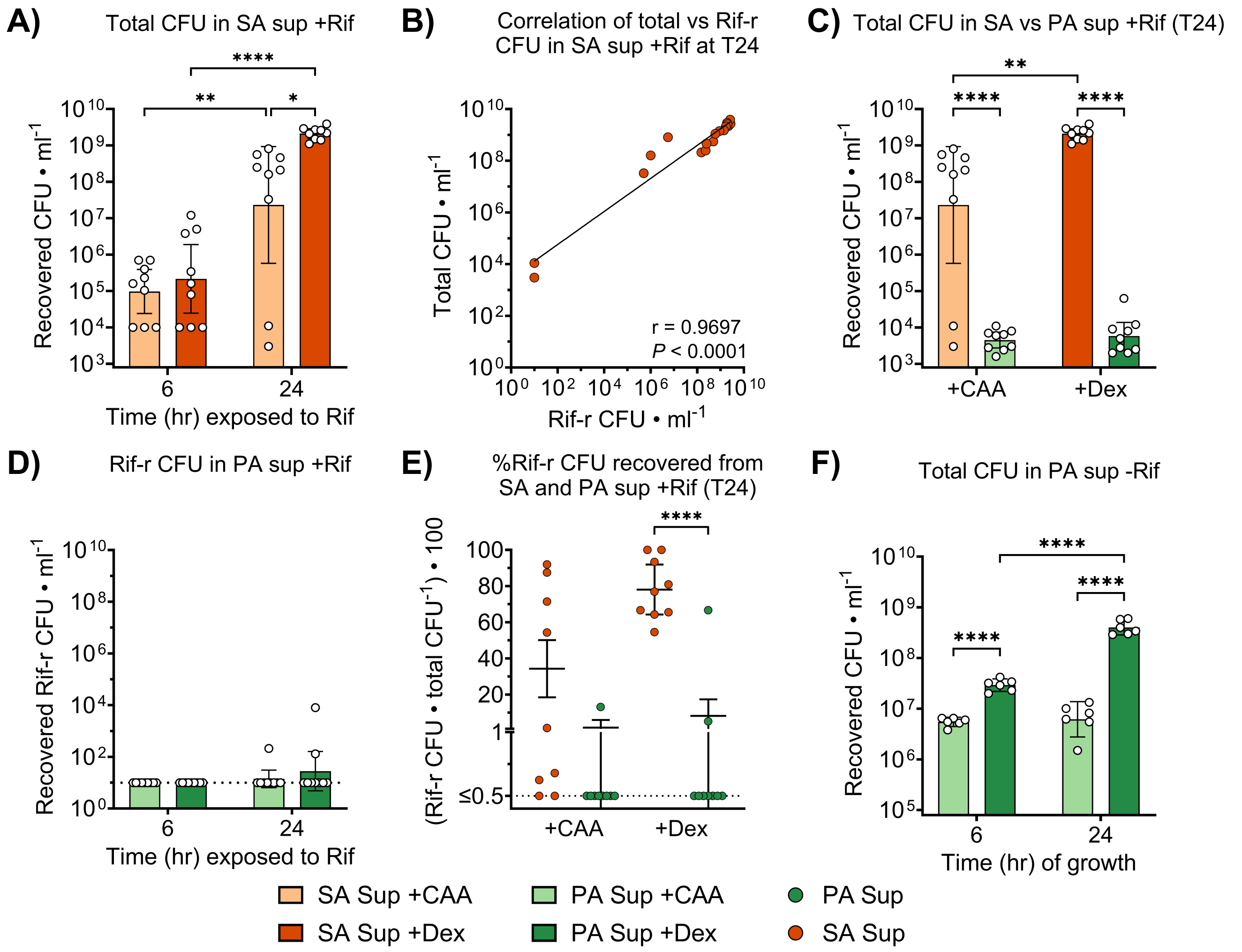

Figure S1. Selected comparisons of total or rifampicin-resistant (Rif-r) *S. aureus* burdens recovered from various supernatant conditions. **(A)** Comparison of total *S. aureus* burdens in Rif-treated *S. aureus* supernatant (SA sup) supplemented with casamino acids (CAA) or dextrose (Dex) at T6 and T24. **(B)** Correlation between total and Rif-r *S. aureus* CFU recovered from Rif-treated SA sup at T24, pooled from samples supplemented both with and without Dex. **(C)** Comparison of total *S. aureus* burdens in Rif-treated SA sup versus *P. aeruginosa* supernatant (PA sup) supplemented with CAA or Dex at T24. **(D)** Comparison of Rif-r *S. aureus* burdens in Rif-treated PA sup supplemented with CAA or Dex at T6 and T24. **(E)** Percentage of Rif-r *S. aureus* in each sample at T24 from SA sup versus PA sup supplemented with CAA or Dex. Shown are means with standard error of the percentage of Rif-r colonies per sample derived from proportions for graphical display. **(F)** Total recovered *S. aureus* from untreated PA sup with CAA or Dex at T6 versus T24. Bars in A, C, D, and F represent geometric means and 95% CI. *$P < 0.05$, **$P < 0.01$, ****$P < 0.0001$, two-way ANOVA with Sidak's multiple comparisons between groups differing by a single experimental factor. Dotted line represents the limit of detection.

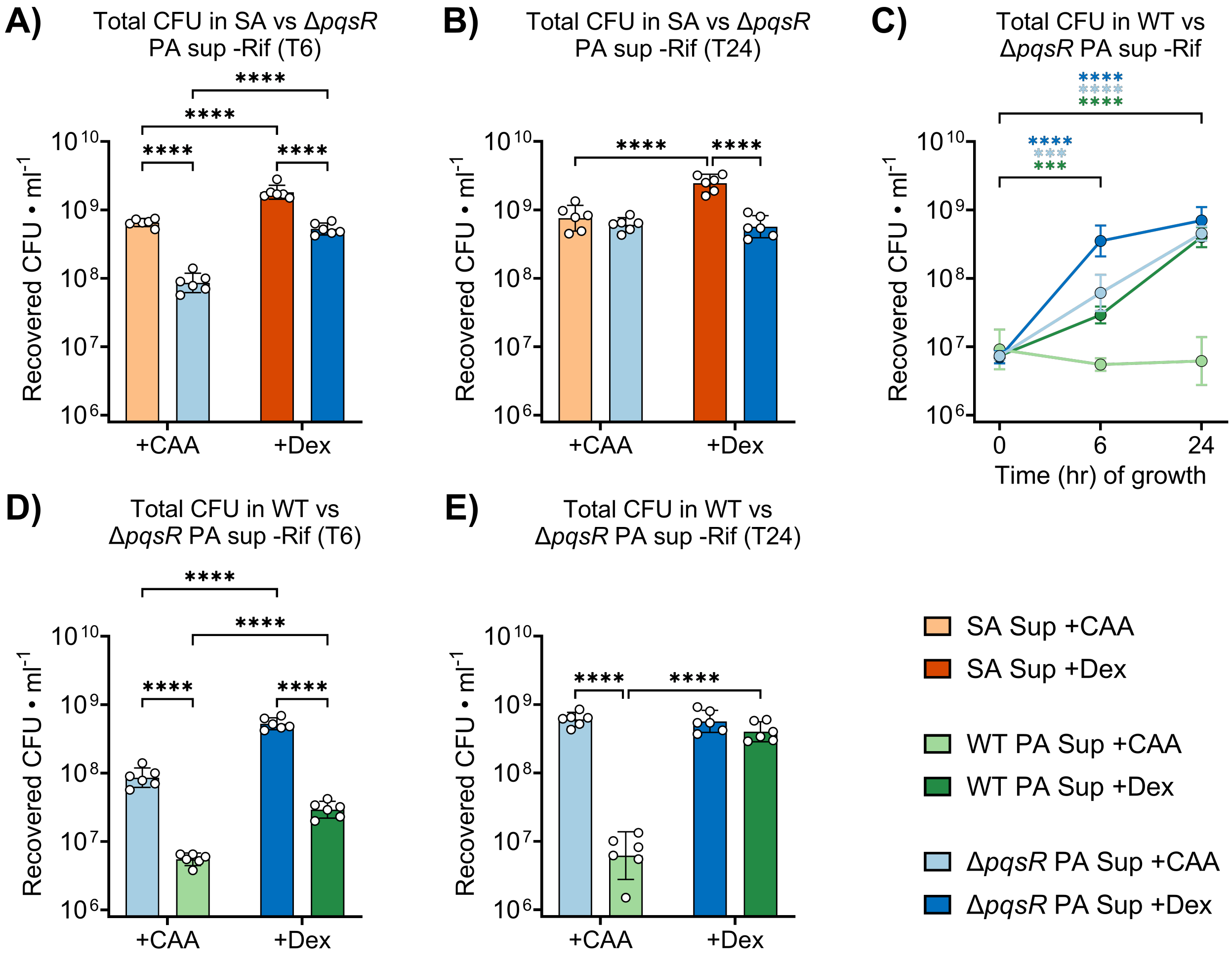

Figure S2. Comparison of total *S. aureus* burdens recovered from Δ*pqsR P. aeruginosa* supernatants (Δ*pqsR* PA sup) versus *S. aureus* supernatants (SA sup) or wild-type *P. aeruginosa* supernatants (WT PA sup) without rifampicin (Rif) treatment. Comparison of total *S. aureus* burden in untreated SA sup versus untreated Δ*pqsR* PA sup at **(A)** T6 and **(B)** T24 with casamino acid (CAA) or dextrose (Dex) supplementation. **(C)** Total *S. aureus* burdens in WT PA sup and Δ*pqsR* PA sup supplemented with CAA or Dex, following 0, 6, and 24 hr of incubation. Comparison of total *S. aureus* burden in WT PA sup versus Δ*pqsR* PA sup at **(D)** T6 and **(E)** T24 with CAA or Dex. Points and bars represent geometric means and 95% CI. ****P* < 0.001, *****P* < 0.0001, two-way ANOVA with Sidak's multiple comparisons between groups differing by a single experimental factor (A, B, D, E) or Dunnet's multiple comparisons to T0 (C).

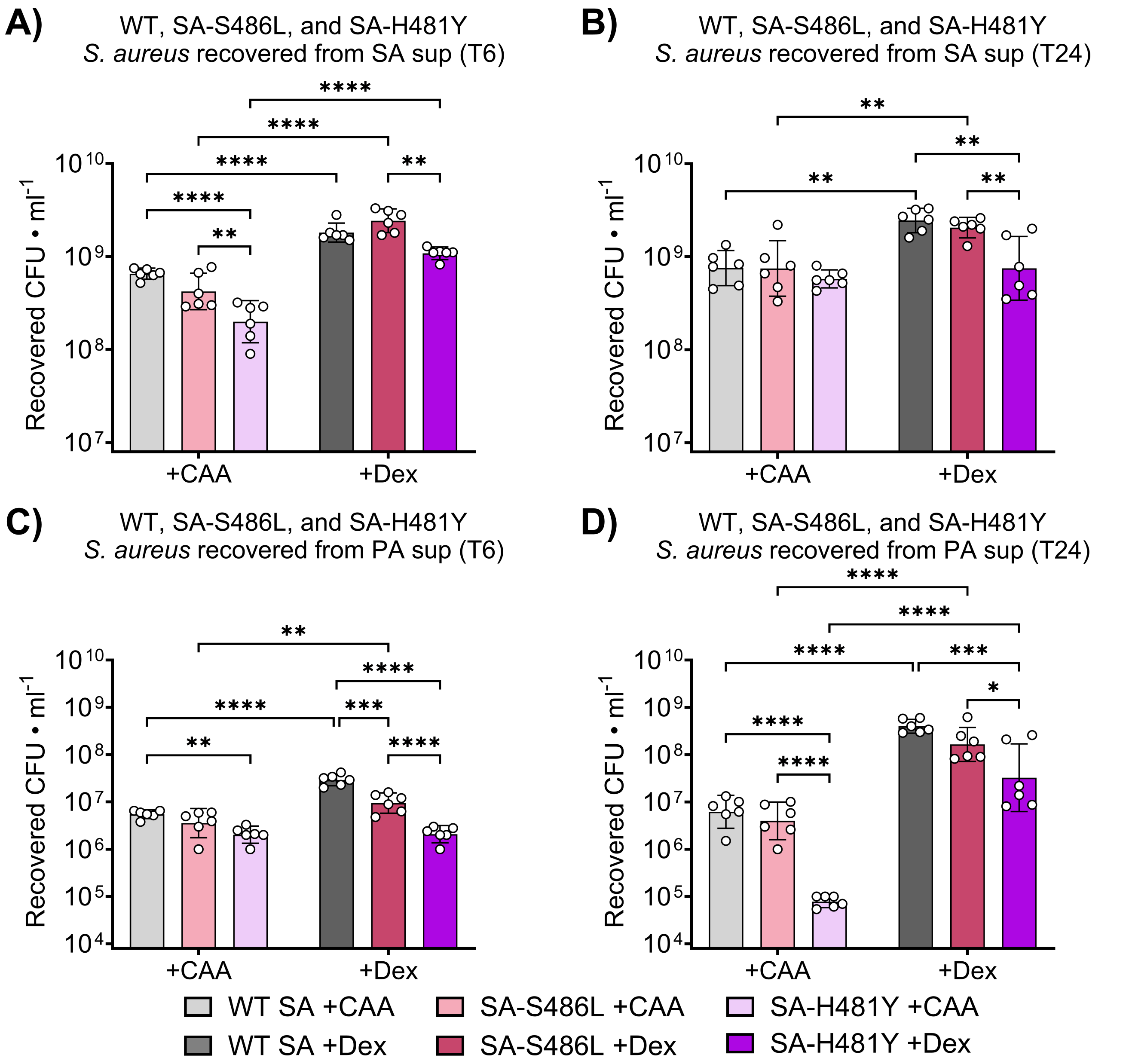

Figure S3. Time point comparisons of WT, SA-S486L, and SA-H481Y *S. aureus* burdens in *S. aureus* supernatants (SA sup) or *P. aeruginosa* supernatants (PA sup). WT, SA-S486L, and SA-H481Y burden was quantified after 6 hr of growth in SA sup supplemented with casamino acids (CAA) or dextrose (Dex) at **(A)** T6 and **(B)** T24. WT, SA-S486L, and SA-H481Y burden was quantified after 6 hr of growth in PA sup supplemented with CAA or Dex at **(C)** T6 and **(D)** T24. Bars represent geometric means and 95% CI. **P* < 0.05, ***P* < 0.01, ****P* < 0.001, *****P* < 0.0001, two-way ANOVA with Sidak's multiple comparisons between groups differing by a single experimental factor.

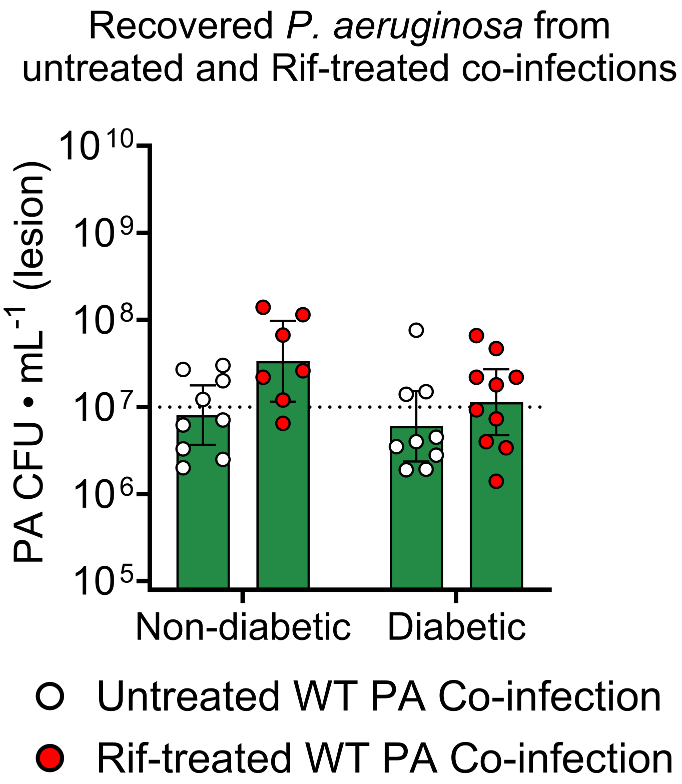

Figure S4. Equivalent *P. aeruginosa* burdens are recovered from untreated and rifampicin (Rif)-treated mice during co-infection with *S. aureus*. Bars represent geometric means and 95% CI. Data were analyzed for significance using two-way ANOVA with Sidak's multiple comparisons between groups differing by a single experimental factor. Dotted line represents infection inoculum.
